# Layoff experience in adulthood and all-cause mortality among US adults, 1979-2022

**DOI:** 10.1093/geroni/igaf122.4375

**Published:** 2025-12-31

**Authors:** Xuexin Yu, Katrina Kezios, Samuel Swift, Kaylie Moropoulos, Adina Zeki Al Hazzouri

**Affiliations:** Columbia University Mailman School of Public Health, New York, New York, United States; Boston University, Boston, Massachusetts, United States; University of New Mexico Health Sciences Center, Albuquerque, New Mexico, United States; Columbia University Medical Center, New York, New York, United States; Columbia University Medical Center, New York, New York, United States

## Abstract

Involuntary job loss due to layoffs is increasingly common in the US, while its association with all-cause mortality remains inconclusive. We investigated the association between cumulative layoff experience over 33 peak-working years in adulthood and mortality during the subsequent 10 years. Data were from 7,234 working-age adults (aged 16+) in National Longitudinal Survey of Youth 1979. Cumulative layoff experience was operationalized as the number of jobs ending in a layoff from 1979-2012: never (n = 5,183), one (n = 1,514), and ≥two layoffs (n = 537). Compared to never being laid-off (n = 5,183), layoff experience contributed to 18.71 (95% CI: 1.09-36.23) and 32.61 (95% CI: 2.66-62.56) excess deaths per 10,000 person-years among participants with one and ≥two layoffs. These estimates correspond to 0.27 and 0.39 excess years of potential life lost per person for one and ≥two layoff groups. In the fully adjusted Cox proportional hazards model, compared to those with no layoffs, individuals with one and ≥two layoffs had 1.23 (95% CI: 1.00 to 1.51) and 1.30 times (95% CI: 0.96 to 1.77) higher hazards of death from 2012-2022. The observed association did not statistically differ by timing of layoffs, sex/gender, and race/ethnicity. Findings suggest job insecurity may be an important determinant of life expectancy.

